# Neural correlates of appreciating natural landscape and landscape garden: Evidence from an fMRI study

**DOI:** 10.1002/brb3.1335

**Published:** 2019-06-01

**Authors:** Wei Zhang, Xianyou He, Sizhe Liu, Ting Li, Jinhui Li, Xiaoxiang Tang, Shuxian Lai

**Affiliations:** ^1^ Guangdong Key Laboratory of Mental Health and Cognitive Science, Center for Studies of Psychological Application School of Psychology South China Normal University Guangzhou P. R. China; ^2^ Key Laboratory of Chinese Learning and International Promotion of South China Normal University Guangzhou P. R. China; ^3^ School of Architecture & State Key Laboratory of Subtropical Building Science, Guangdong Engineering & Technology Research Center for Modern Architecture Design South China University of Technology Guangzhou P. R. China; ^4^ Preschool Education Guidance Center of Tianhe District Public Kindergarten of Guangzhou Government Guangzhou P. R. China

**Keywords:** appreciation, fMRI, landscape garden, natural landscape, naturalness and artificiality of landscapes

## Abstract

**Introduction:**

Rambling natural landscapes or landscape gardens may invoke positive emotions. However, the manner in which people experience landscape gardens and the cortical differences in the appreciation of the naturalness and artificiality of landscapes remain unknown.

**Methods:**

This study scanned participants with functional magnetic resonance imaging while they viewed photographs of natural landscapes and landscape gardens and performed scene type judgment task.

**Results:**

As predicted, we identified brain regions that were associated with perceptual process, cognitive process, and rewarding experience when appreciating natural landscapes and landscape gardens without color preference. Meanwhile, the contrast between the appreciation of landscape gardens and natural landscapes was characterized by stronger activations of the inferior occipital lobe, the left superior parietal lobule (SPL), the right fusiform gyrus, the right cuneus, and the right hippocampus.

**Conclusions:**

Responses in these regions indicate that the appreciation of landscape gardens and natural landscapes relies on common cortical regions, and suggest the possibility that the inferior occipital lobe, the SPL, the fusiform gyrus, and the cuneus may be specifically associated with the appreciation of landscape gardens.

## 1 INTRODUCTION

Human beings worldwide are surrounded by various architectural spaces (Evans & McCoy, 1998). The manner in which our brain and mind experience architectural settings is an interesting issue worth considering in the neuroscience of architecture (Eberhard, 2009). Functional neuroimaging evidence of architectural space has identified the brain activations accompanying the perception of architectural parameters, such as visual features, spatial configurations and architectural styles, including the occipital place areas, the superior and the middle temporal gyri (MTG), the precuneus, and the fusiform gyrus. The occipital place areas respond to perceptual information regarding architecture (Marchette, Vass, Ryan, & Epstein, 2015; Mégevand et al., 2014), and the superior and the MTG are involved in the motion required for visuo‐spatial exploration (Vartanian et al., 2015). The fusiform gyrus contains neural representations of architectural styles (Choo, Nasar, Nikrahei, & Walther, 2017).

Moreover, Dance (2017) further proposed that the processing of architecture and space is not only related to the processing of perception, but is also associated with emotional processing and experience. Previous behavioral empirical studies have also found that contact with the natural environment or green space in urban areas is in a sense good for the experience of human beings, in terms of stress reduction (Joye, 2007), greater vitality (Ryan et al., 2010), higher rewarding experience (Zhang, Tang, He, & Chen, 2018; Zhang, Tang, He, & Lai, 2018), an improvement in social performance (Zhang, Piff, Iyer, Koleva, & Keltner, 2014) and cognitive function (Atchley, Strayer, & Atchley, 2012; Bratman, Hamilton, & Daily, 2012). This evidence coincides with the framework of evolutionary psychology, which argues that the perception and appreciation of architectural environments or scenes with green space is associated with survival, reproduction, and environmental adaptations of humans (Killin, 2013; Seghers, 2015; Zhang, Tang, He, & Lai, 2018), and is linked to the enhancement of positive experience.

Regarding the neural basis of the emotional experience during the perception and appreciation of architectural spaces, Kirk and colleagues' pioneering work has demonstrated that the processing of reward during architecture appraisal is associated with the medial orbitofrontal cortex (mOFC) and subcallosal cingulate gyrus (Kirk, Skov, Christensen, & Nygaard, 2009). Researchers also found that enclosed architectural spaces elicit more avoidance decisions and are linked to the activation of the cingulate gyrus, which is related to emotional processes (Vartanian et al., 2015). The perception and appreciation of curvilinear architectural spaces is also associated with the anterior cingulate cortex (ACC) activity, which is responsive to reward and emotional processing (Vartanian, Navarrete, et al., 2013). This neuroimaging evidence is consistent with the theoretical model of architectural appreciation, which suggests that there are three systems contributing to the perception and appreciation of architecture. The sensorimotor system is related to sensory processing (e.g., visual perception) and motor responses (e.g., visuo‐spatial exploration and motivation for approaching or avoiding a structure), the knowledge‐meaning system conducts signals pertaining to personal experience and individual differences during processing, and the emotion‐valuation system generates feelings, emotions, and rewarding experience (Coburn, Vartanian, & Chatterjee, 2017). As landscape gardens (e.g., the landscape gardens of Suzhou, China) constitute a type of architecture that is composed of natural elements (Zhang, Tang, He, & Chen, 2018), the manner in which people experience landscape gardens and the cortical differences between the perception and appreciation of natural landscapes (naturalness of landscapes) and landscape gardens (artificiality of landscapes) are two core questions addressed in this study.

Using functional magnetic resonance imaging (fMRI), we scanned participants when they viewed landscape gardens (LGO) and natural landscapes (NLO) photographs. A scene type judgment task (i.e., indicating whether the color photograph was a landscape garden or a natural landscape) was adopted to investigate the cortical differences between the perception and appreciation of natural landscapes (naturalness of landscapes) and landscape gardens (artificiality of landscapes). In addition, the lower definition versions of each original photograph of the landscape garden (LGL) and the natural landscape (NLL) were created and served as the baseline because they retained only the general colors and outlines of the original photographs, and could eliminate the effect of color preference (Vartanian & Goel, 2004; Wang, Mo, Vartanian, Cant, & Cupchik, 2015; Zhang, Tang, He, & Lai, 2018).

The contrast of “LGO>NLO” was expected to reveal the neural differences in the perception and appreciation of landscape gardens and natural landscapes, and the preference for color could be eliminated by the contrast of “LGO>LGL” and “NLO>NLL.” We hypothesized that the appreciation of landscape gardens and natural landscapes might rely on similar neural pathways involved in the appreciation of other visual objects, including the brain regions associated with perceptual, cognitive, emotional, and reward processing (Chatterjee, 2011; Chatterjee & Vartanian, 2014). In addition, we expected that some of the brain regions related to visuo‐spatial processing and architectural parameters would be activated in the contrast between landscape gardens and natural landscapes, which could provide preliminary evidence for the cortical differences in the perception of the naturalness and artificiality of landscapes.

## 2 EXPERIMENTAL PROCEDURE

### 2.1 Participants

Sixteen right‐handed college‐aged participants between 18 and 24 years of age (mean age = 20.50 years, *SD* = 1.71) were recruited and paid for their participation. None of the participants had any previous training in art or architecture, and none of them had a history of neurological or psychiatric disorders. They all had normal or corrected‐to‐normal vision and normal color vision. Prior to the experiment, participants signed an informed consent form. The protocol was approved by the Institute Ethics Committee of South China Normal University, and the study was designed and conducted in accordance with the Declaration of Helsinki.

### 2.2 Experimental procedures

#### 2.2.1 Materials

Thirty‐six color photographs of landscape gardens and 36 color photographs of natural landscapes were selected from the public archive at http://image.baidu.com/ (an original set of 200 color photographs), using a prior behavioral rating obtained from a separate group of participants (*n* = 31, none of them had any special training in art or architecture) based on (a) the aesthetic quality, (b) the complexity, and (c) the familiarity of the photographs on a 5‐point scale. The rating results of the two sets of materials showed no significant differences in terms of aesthetic quality (3.95 ± 0.43 and 3.92 ± 0.51, for landscape gardens and natural landscapes colored photographs, respectively), *F* (1, 30) = 0.11, *p* = 0.743, *ƞ* ^2^ = 0.01, complexity (3.32 ± 0.45 and 3.19 ± 0.56, for landscape gardens and natural landscapes colored photographs, respectively), *F* (1, 30) = 1.71, *p* = 0.201, *ƞ* ^2^ = 0.05, and familiarity with the photographs (1.30 ± 0.37 and 1.24 ± 0.33, for landscape gardens and natural landscapes colored photographs, respectively), *F* (1, 30) = 1.55, *p* = 0.223, *ƞ* ^2^ = 0.05.

A lower definition version for the color photographs of each landscape garden and natural landscape was created in Adobe Photoshop CS6. The lower definition versions of the photographs of the landscape gardens and natural landscapes served as the baseline because they retained only the general colors and outlines of the original photographs.

**Figure 1:**
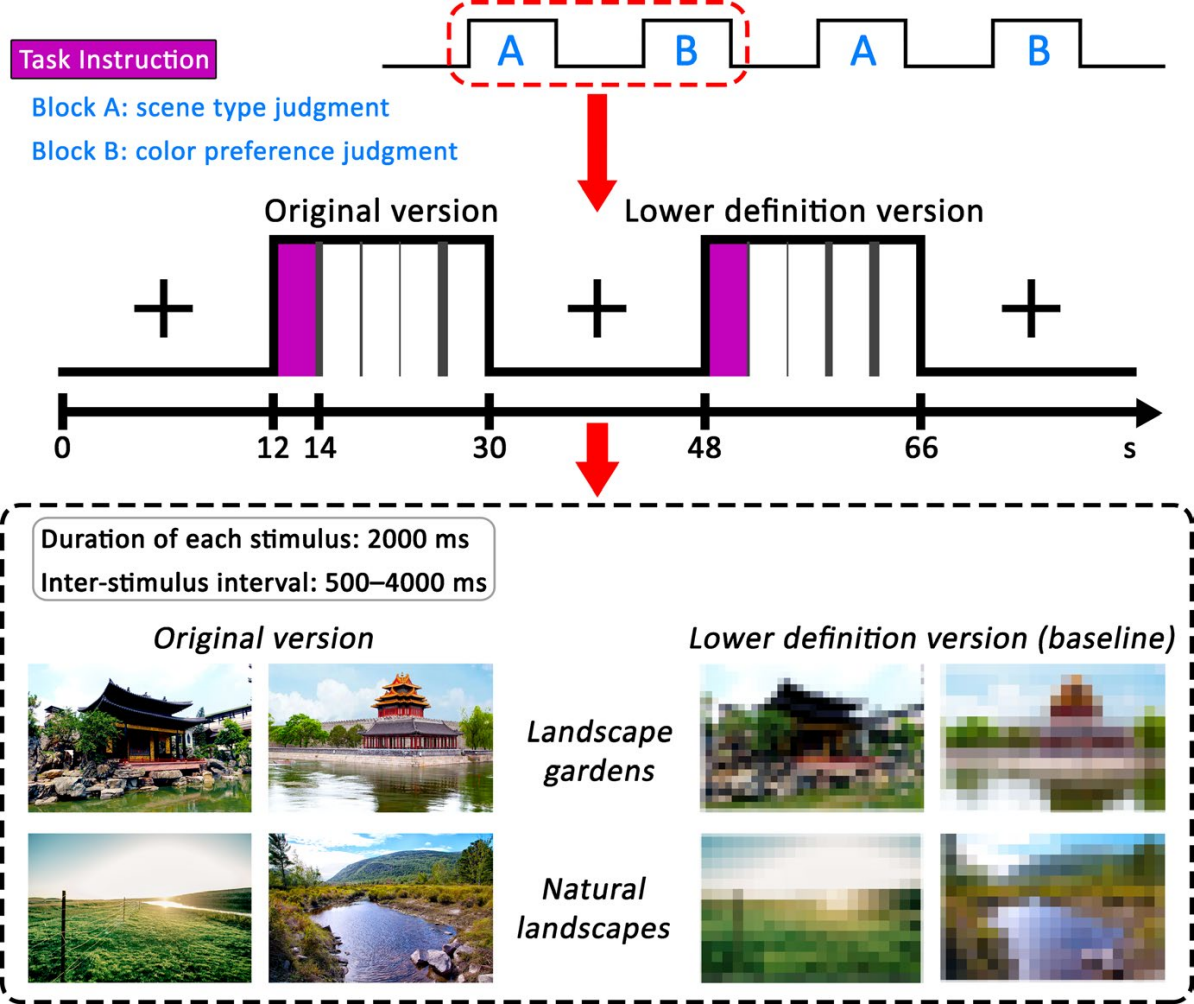
Experimental design, procedure and examples of stimuli. Two types of tasks were performed in separate blocks, namely, the scene type judgment (landscape garden vs. natural landscape) and the color preference judgment (baseline condition; like vs. dislike). In this figure, we used photographs taken by the authors instead of examples of the stimuli used in the scanning experiment due to licensing restrictions

All the experimental materials were adjusted to the same size within a rectangular “window” that was 300 × 200 pixels, centred on a 600 × 400 pixel black background and presented at a screen resolution of 800 × 600 pixels. Samples of the materials and the experimental procedures for the present study are illustrated in Figure 1.

#### 2.2.2 Procedure and tasks

In the fMRI scanner, the trials were presented according to a hybrid design. The experimental session had 18 blocks pertaining to each of the two versions, namely, the original version and the lower definition version (baseline). The original version was used for 36 LGO trials and 36 NLO trials, and the lower definition version was used for 36 LGL trials and 36 NlL trials. Participants underwent three separate scanning runs; each run consisted of 12 blocks. The block order was fixed and counterbalanced across participants. The instructions for the visual task appeared for 2,000 ms before the onset of each block. Each block contained four trials and lasted for 18 s, followed by 18 s of fixation. Within each block, each trial was conducted for 2,000 ms, including the response time, in pseudo‐random order (event‐related design) and was followed by a jittered 500–4,000 ms inter‐stimulus interval.

During the experimental session, participants needed to perform two kinds of tasks. For the scene type judgment task, participants were required to observe the image and indicate whether the color photograph was a landscape garden or a natural landscape by pressing one of two buttons; one button was held in the left hand, whereas the other button was held in the right hand. For the color preference judgment task, participants were shown the lower definition versions of the photographs and instructed to respond whether they liked the color of each photograph by pressing one of the two buttons. The finger‐response mapping was fixed and counterbalanced across participants.

After scanning, each participant provided a rating for the familiarity he/she had with the color photographs of landscape gardens and natural landscapes to further rule out the effect of familiarity on the results of the scans. The procedures were identical to those used in the prior behavioral rating trial.

### 2.3 Data acquisition

Imaging data were acquired with a 3‐Tesla Siemens Trio Tim MRI scanner fitted with a 12‐channel phased‐array head coil at the Brain Imaging Centre of South China Normal University. Changes in blood oxygen level dependence were obtained from the T2*‐weighted gradient echo, echo‐planar imaging (EPI) sequence (TR = 2000 ms; TE = 30 ms; flip angle = 90°; FOV = 224 mm; matrix size = 64 × 64). Each volume consisted of 32 axial slices, with a slice thickness of 3.5 mm and an inter‐slice gap of 0.8 mm; the whole brain was covered. T1‐weighted anatomical images were acquired with 3‐D magnetization prepared rapid gradient echo (MP‐RAGE) sequence (TR = 1900 ms; TE = 2.52 ms; flip angle = 9°; voxel size = 1 × 1 × 1 mm) from each participant at the end of the experimental session.

### 2.4 Data analysis

Imaging data were preprocessed using SPM8 (http://www.fil.ion.ucl.ac.uk/spm/) implemented in MATLAB with the extension software Data Processing and Analysis of Brain Imaging (DPABI, http://rfmri.org/dpabi, Yan, Wang, Zuo, & Zang, 2016). The preprocessing steps included correction for slice‐scan timing and head motion artifacts. The EPI images were realigned spatially, normalized to the MNI space, resampling at a voxel size of 3 × 3 × 3 mm^3^, and spatially smoothed with an isotropic 6‐mm full width‐half‐maximum Gaussian kernel. One participant was excluded from the subsequent analysis because the individual image had a >2 mm maximum displacement and >1.5° rotation.

In the single‐participant GLM, the stimulus functions were convolved with a canonical hemodynamic response function. We modeled the following four regressors of interest: LGO, NLO, LGL, and NLL. Head movement parameters calculated from the realignment preprocessing step were included as regressors that were not of interest. A high‐pass filter with a cut‐off period of 128 s was used to remove low‐frequency drift terms. At the first level, analyses were performed individually for each participant. Contrasts images between each experimental condition and the baseline condition were created, and were subsequently entered into the second‐level analysis treating participants as a random factor and modelled the data using Flexible Factorial analyses.

On the basis of the contrasts, we computed a conjunction analysis between “LGO>LGL” and “NLO>NLL” to identify areas of brain activation common to the appreciation of landscape garden and natural landscape using the minimum statistic approach (Nichols, Brett, Andersson, Wager, & Poline, 2005). Effect sizes in the contrasts of “LGO>LGL,” “NLO>NLL,” and “LGO>NLO” were estimated based on the mean values of the regions of interest (ROI). ROI was extracted in the four regressors for each participant, and was defined using a sphere with 6 mm radius centred on the peak voxel.

## 3 RESULTS

### 3.1 Behavioral results

#### 3.1.1 Accuracy of scene type judgment

The accuracy of the participants' scene type judgments was submitted to one‐way repeated‐measures ANOVA, with the subjects as a random effect. The overall accuracy was very high (0.95 ± 0.05 vs. 0.95 ± 0.06 for the original versions of landscape gardens and natural landscapes, respectively) and revealed no significant difference between the two sets of materials, *F* (1, 15) = 0.02, *p* = 0.882, *ƞ* ^2^ < 0.01.

#### 3.1.2 Postscan ratings of familiarity

For the mean postscan ratings of familiarity, no significant difference was found between the original versions of the landscape gardens (1.42 ± 0.72) and the natural landscapes (1.25 ± 0.44), *F* (1, 15) = 2.27, *p* = 0.152, *ƞ* ^2^ = 0.13), suggesting that the effect of familiarity with the two sets of materials can be eliminated.

### 3.2 fMRI results

#### 3.2.1 Brain regions for appreciating landscape gardens without color preference

**Figure 2:**
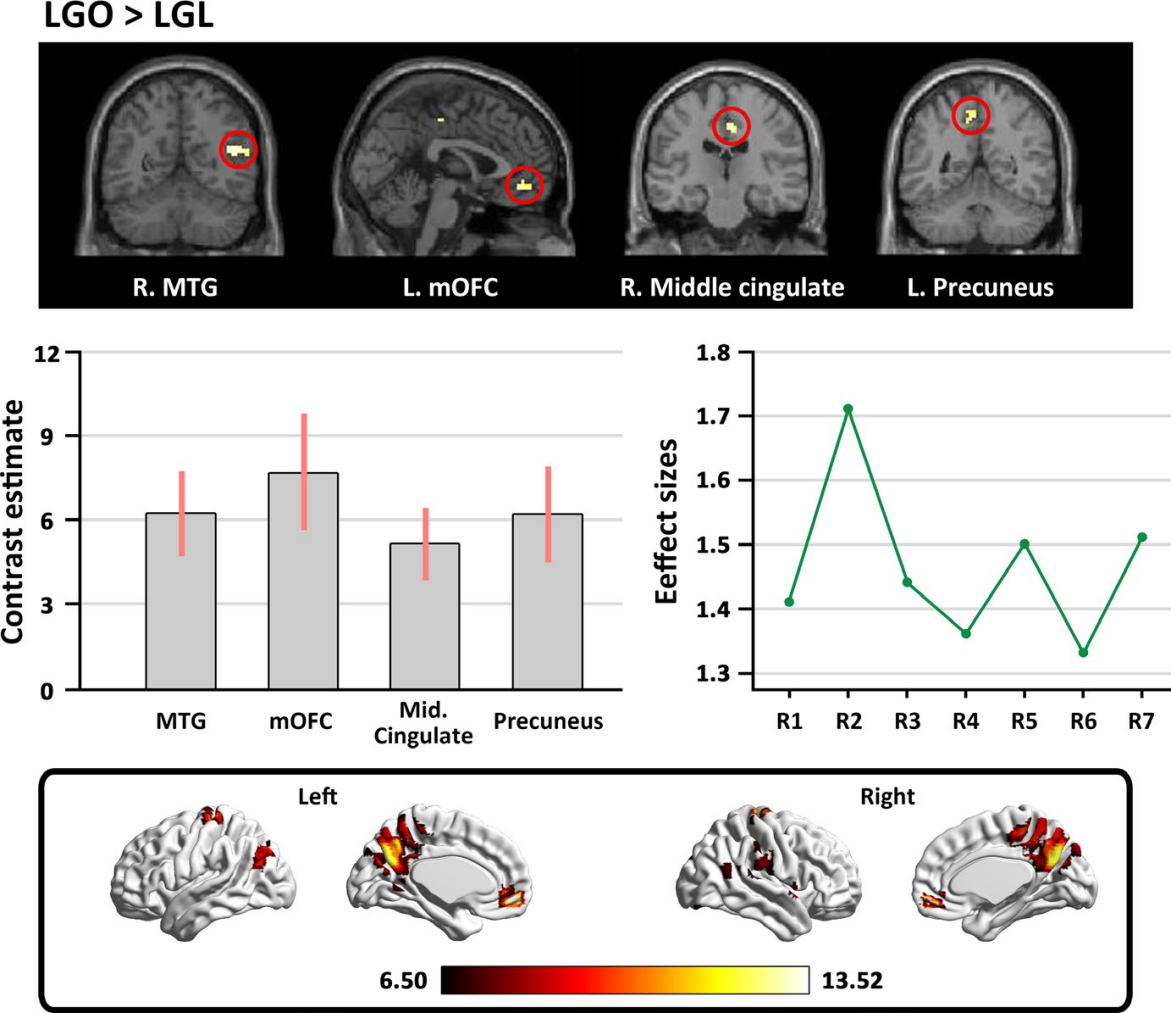
The main cerebral areas involved in appreciating landscape gardens without color preference. The labels from R1 to R7 in “Effect sizes” refer to: R1 = the left MTG (1.41), R2 = the right MTG (1.71), R3 = the right STG (1.44), R4 = the left SFG (1.36), R5 = the right middle cingulate (1.50), R6 = the left mOFC (1.33), R7 = the left precuneus (1.51). Effect sizes are indicated in parenthesis. mOFC, medial orbitofrontal cortex; MTG, middle temporal gyri; SFG, superior frontal gyrus; STG, superior temporal gyrus

To identify the brain regions sensitive to the appreciation of landscape gardens without color preference during the scene type judgment task, the “LGO>LGL” contrast was examined. We observed stronger activation in the bilateral MTG, the right superior temporal gyrus (STG), the left superior frontal gyrus (SFG), the right middle cingulate, the left mOFC, and the left precuneus (see Table 1 and Figure 2).

**Table 1:** Activated areas for appreciating landscape gardens without color preference

Brain regions	Hemisphere	Peak coordinates			*t*‐Score	Cluster size
		*x*	*y*	*z*		
Original version of landscape gardens > Lower definition version of landscape gardens						
MTG	L	−51	3	−21	5.73	8
	R	45	−57	18	6.99	23
STG	R	54	−36	15	6.27	9
SFG	L	−21	27	39	6.24	7
Middle cingulate	R	6	−21	42	6.80	25
Medial OFC	L	−1	45	−12	6.17	20
Precuneus	L	−6	−51	51	6.05	13

Note

Coordinates refer to the stereotactic space of the Montreal Neurological Institute. The activations are FWE corrected at the voxel level and cluster level (*p* < 0.05).

Abbreviations: MTG, middle temporal gyri; medial OFC, medial orbitofrontal cortex; SFG, superior frontal gyrus; STG, superior temporal gyrus.

#### 3.2.2 Brain regions for appreciating natural landscapes without color preference

**Figure 3:**
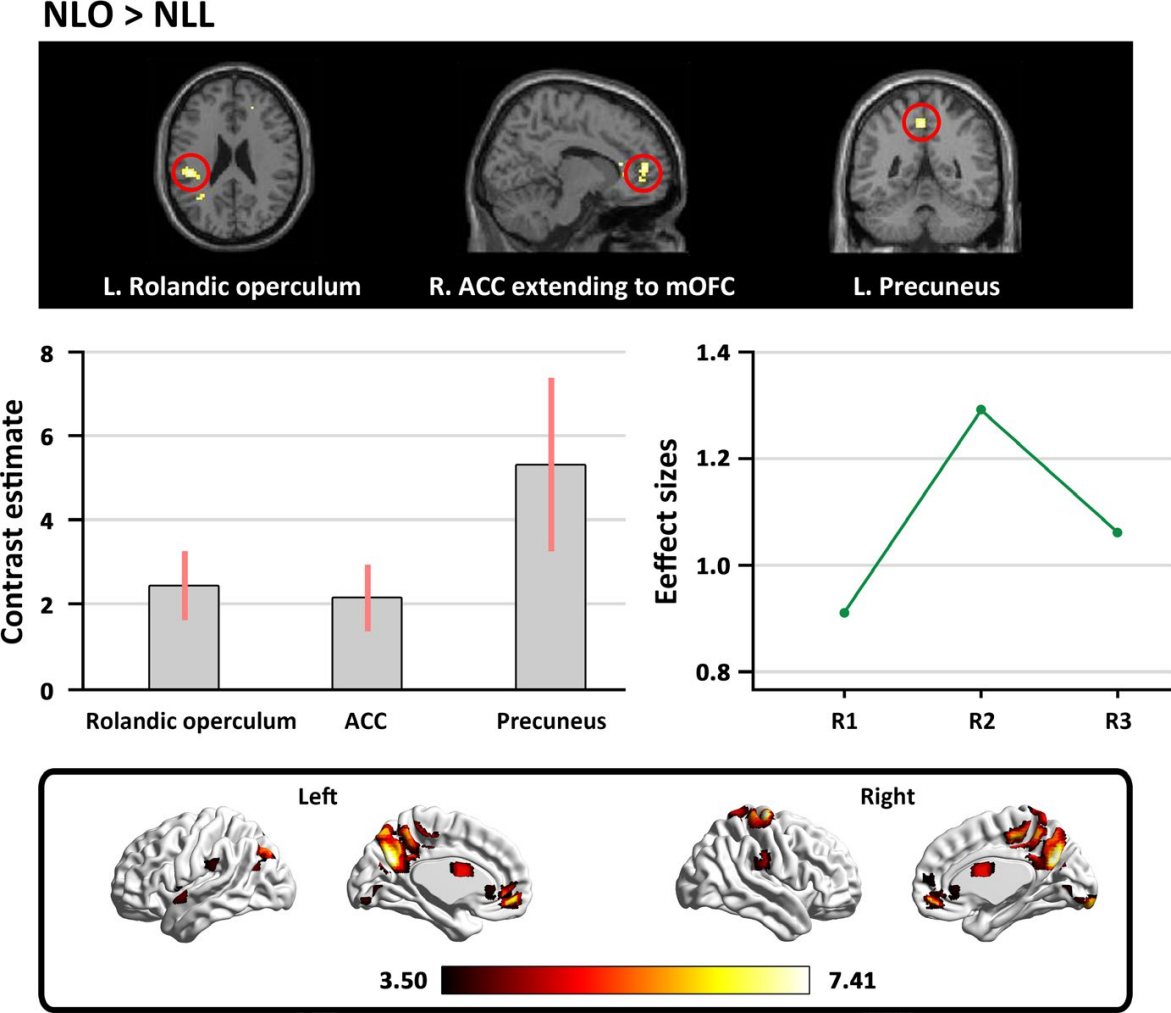
The main cerebral areas involved in appreciating natural landscapes without color preference. The labels from R1 to R3 in “Effect sizes” refer to: R1 = the right ACC extending to the right mOFC (0.91), R2 = the left rolandic operculum (1.29), R3 = the left precuneus (1.06). Effect sizes are indicated in parenthesis. ACC, anterior cingulate cortex; mOFC, medial orbitofrontal cortex

In the contrast of “NLO>NLL,” we found that appreciation of natural landscape without color preference was associated with activity in the right ACC extending to the right mOFC, the left rolandic operculum, and the left precuneus (see Table 2 and Figure 3).

**Table 2:** Activated areas correlating with the appreciation of natural landscapes without color preference

Brain regions	Hemisphere	Peak coordinates			*t*‐Score	Cluster size
		*x*	*y*	*z*		
Original version of natural landscapes > Lower definition version of natural landscapes						
ACC extending to the right medial OFC	R	12	51	9	4.44	37
Rolandic operculum	L	−45	−30	24	4.58	29
Precuneus	L	−3	−48	51	4.31	18

Note

Coordinates refer to the stereotactic space of the Montreal Neurological Institute. The statistical significance refers to *p* < 0.001 at voxel level (uncorrected), *p* < 0.05 at cluster level (FWE corrected).

Abbreviations: ACC, anterior cingulate cortex; medial OFC, medial orbitofrontal cortex.

#### 3.2.3 Brain regions revealed by conjunction analysis

**Figure 4:**
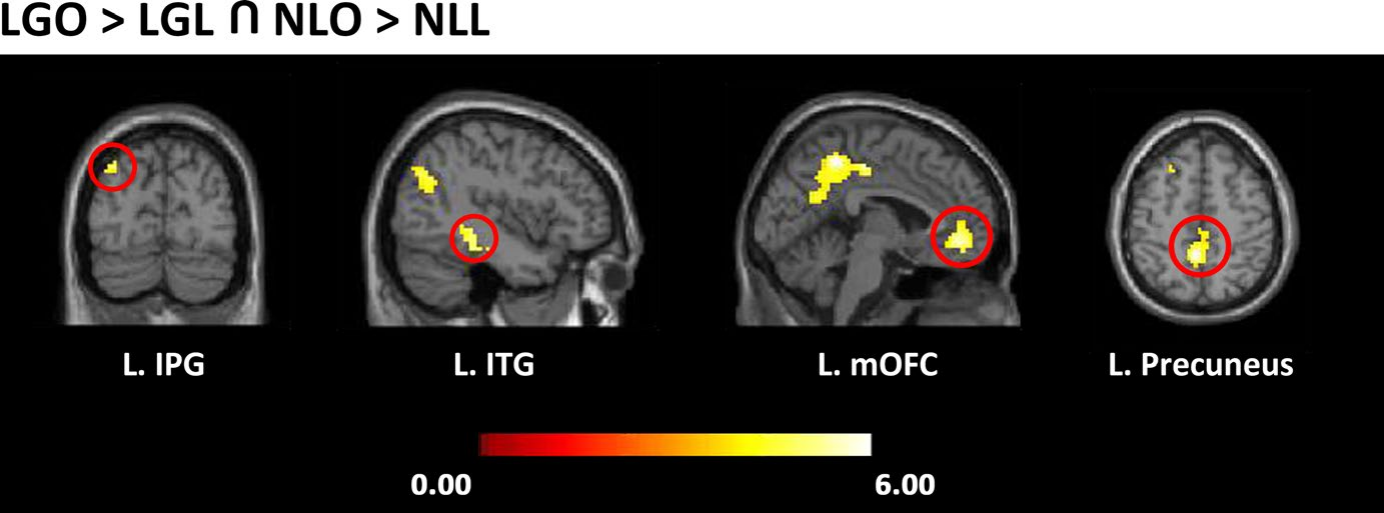
The conjunction results of the appreciation of natural landscapes and landscape gardens

A conjunction analysis was performed with the comparisons between “LGO>LGL” and “NLO>NLL” to identify the common regions activated when viewing landscape gardens and natural landscapes. The results showed that the left precuneus extending to the left middle cingulate, the left mOFC extending to the right ACC, the left inferior temporal gyrus (ITG) extending to the left hippocampus, and the left inferior parietal gyrus were commonly activated (see Table 3 and Figure 4).

**Table 3:** Activated areas in the conjunction analysis between natural landscapes and landscape gardens

Brain regions	Hemisphere	Peak coordinates			*t*‐Score	Cluster size
		*x*	*y*	*z*		
Conjunction of the appreciation of natural landscapes and landscape gardens						
Precuneus (extending to middle cingulate)	L	−3	−48	51	5.45	447
Medial OFC (extending to the right ACC)	L	−3	45	−9	5.07	162
ITG (extending to hippocampus)	L	−42	−36	−12	4.70	66
IPG	L	−36	−78	42	4.30	85

Note

Coordinates refer to the stereotactic space of the Montreal Neurological Institute. The statistical significance refers to *p* < 0.001 at voxel level (uncorrected), *p* < 0.05 at cluster level (FWE corrected).

Abbreviations: ACC, anterior cingulate cortex; IPG, inferior parietal gyrus; ITG, inferior temporal gyrus; medial OFC, medial orbitofrontal cortex.

#### 3.2.4 Cortical differences between the appreciation of landscape gardens and natural landscapes

**Figure 5:**
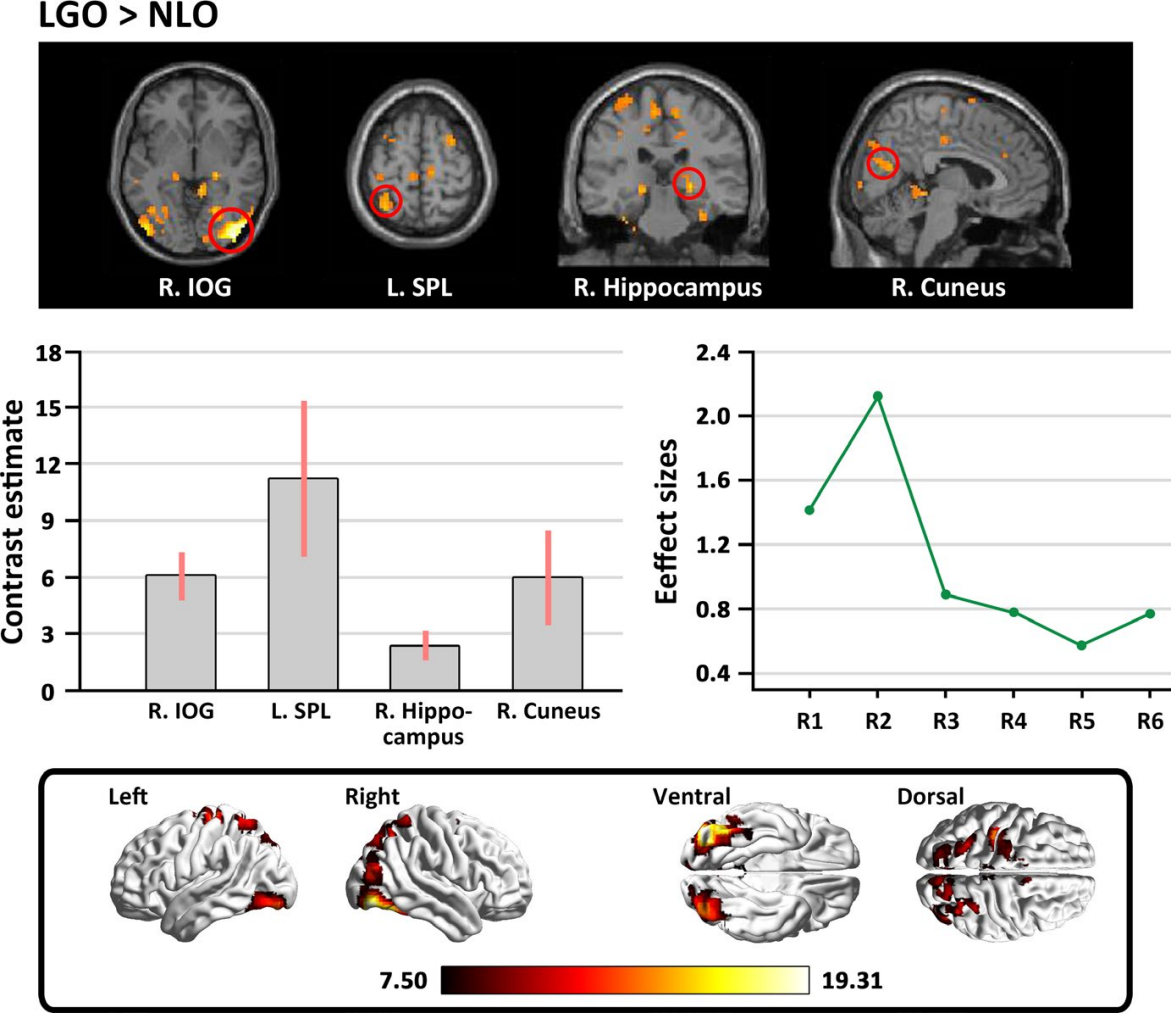
The main cerebral areas involved in the analysis of variance between natural landscapes and landscape gardens. The labels from R1 to R6 in “Effect sizes” refer to: R1 = left IOG extending to the MOG (1.41), R2 = right IOG extending to the fusiform gyrus (2.12), R3 = the right hippocampus (0.88), R4 = the right cuneus (0.77), R5 = the left SPL (0.56), R6 = the SMA (0.76). Effect sizes are indicated in parenthesis. IOG, inferior occipital gyrus; MOG, middle occipital gyrus; SMA, supplementary motor area; SPL, superior parietal lobule

The contrast between the original versions of the landscape gardens and the natural landscapes (LGO>NLO) revealed stronger activation in response to the original version of the landscape gardens in the left inferior occipital gyrus (IOG) extending to the middle occipital gyrus, the right IOG extending to the fusiform gyrus, the right hippocampus, the right cuneus, the left superior parietal lobule (SPL), and the supplementary motor area (SMA). However, no significant activation was found in the opposite comparison (see Table 4 and Figure 5).

**Table 4:** Activated areas of the analysis of variance between landscape gardens and natural landscapes

Brain regions	Hemisphere	Peak coordinates			*t*‐Score	Cluster size
		*x*	*y*	*z*		
Original version of landscape gardens > Original version of natural landscapes						
IOG (extending to MOG)	L	−27	−84	−9	7.71	900
IOG (extending to fusiform gyrus)	R	39	−87	−3	7.87	1,200
Hippocampus	R	24	−27	−4	4.90	63
Cuneus	R	6	−75	21	4.83	123
SPL	L	−33	−57	63	4.61	108
SMA	R	3	0	48	4.43	172
Original version of natural landscapes > Original version of landscape gardens						
Nonsignificant						

Note

Coordinates refer to the stereotactic space of the Montreal Neurological Institute. The statistical significance refers to *p* < 0.001 at voxel level (uncorrected), *p* < 0.05 at cluster level (FWE corrected).

Abbreviations: IOG, inferior occipital gyrus; MOG, middle occipital gyrus; SMA, supplementary motor area; SPL, superior parietal lobule.

## 4 DISCUSSION

This study aimed to identify the neural basis underlying the perception and appreciation of natural landscapes and landscape gardens by subtracting the regions activated by the baseline condition (the lower definition versions of the photographs of natural landscapes and landscape gardens) from the regions of the brain activated by viewing color photographs of natural landscapes and landscape gardens. Meanwhile, subtracting the results obtained by viewing color photographs of natural landscapes from those obtained when viewing the photographs of landscape gardens allowed us to identify the cortical differences in the perception and appreciation of landscape gardens (artificiality of landscapes) and natural landscapes (naturalness of landscapes).

In line with our hypothesis, by controlling color preference through the baseline condition, we found stronger activation of the bilateral MTG, right STG, left SFG, right middle cingulate, left mOFC, and left precuneus prompted by viewing the original version of landscape garden photographs. It has been established that the MTG and the STG are involved in perceiving motion in an open space (Vartanian et al., 2015), and that the MTG also signals individual differences during the perception and appreciation of visual objects (Vartanian, Goel, Lam, Fisher, & Granic, 2013). Again, the precuneus has been found to be involved in art appreciation and the expectation of reward (Doñamayor, Schoenfeld, & Münte, 2012; Mizokami et al., 2014). The mOFC has been strongly implicated in rewarding emotional experience of visual and musical stimuli (Ishizu & Zeki, 2011, 2013, 2017; O'Doherty et al., 2003; Zeki, Romaya, Benincasa, & Atiyah, 2014). Given the strong associations between these two brain regions and rewarding emotional experience, our results suggested that perceiving landscape gardens is rewarding.

Taken together, the involvement of these brain regions supported our hypothesis that the perception and appreciation of landscape gardens may rely on the common neural areas that are active in the perception and appreciation of other visual stimuli, including the combined participation of visual perceptual processing, cognitive processing, and rewarding emotional experience (Berlyne, 1971; Cupchik, 2002; Wang, Mo, Mo, et al., 2015; Zhang et al., 2017; Zhang, Lai, He, Zhao, & Lai, 2016); this finding may support the framework of experiencing art to the neuroscience underlying our perception and appreciation of architecture (Shimamura, 2013), suggesting that the perception and appreciation of architecture engage the sensorimotor, knowledge‐meaning, and emotion‐valuation systems (Chatterjee, 2013; Coburn et al., 2017).

Similar to the findings regarding the perception and appreciation of landscape gardens, the appreciation of the natural landscape activated the left rolandic operculum, the right ACC extending to the right mOFC, and the left precuneus. Moreover, the conjunction analysis between landscape gardens and natural landscapes also revealed that the attentional orienting region, the ventral visual system (ITG; Rushworth, Krams, & Passingham, 2001; Wang, Mo, Mo, et al., 2015), and the processing of appreciation and reward circuitries (precuneus and mOFC) were activated. The results indicate that there are common cortical regions involved in the perception and appreciation of both landscape gardens and natural landscapes.

More importantly, we also investigated the comparison of “LGO>NLO” to identify whether there are neural differences in the activation stimulated by the appreciation of landscape gardens and natural landscapes. As expected, we found stronger activation when viewing the original versions of the landscape garden photographs in the inferior occipital lobe, the left SPL, the right fusiform gyrus, the right hippocampus, the right cuneus, and the SMA. Activities in the inferior occipital lobe have been reported to be more sensitive to the processing of the configuration and shapes of objects (Di Dio, Macaluso, & Rizzolatti, 2007), and activity in the SPL has been linked to visuo‐spatial coding (Di Dio & Gallese, 2009). The fusiform gyrus has been suggested as the region responsible for processing architectural styles (Choo et al., 2017). It is noteworthy that the perception and appreciation of landscape gardens also engaged the right cuneus and hippocampus; past studies have shown that the cuneus is responsive to the appreciation of representational materials (Mizokami et al., 2014), and the activity of the hippocampus correlates with the consolidation of a tendency toward a preference into a firm decision (Ito et al., 2014). These cortical regions imply that the specific activations for appreciating landscape gardens may be linked to multiple layers of processing, including the perception of the global configuration and layout of architecture, the recognition of the architectural style and motif, the rewarding emotional experience, and the embodied motivation to approach the structure (Vartanian et al., 2015; Zhang et al., 2016, 2017).

In conclusion, the present findings support our hypothesis that the appreciation of landscape gardens and natural landscapes relies on the similar neural pathways used to perceive other visual objects, including the combined participation of the visual perceptual process, the cognitive process, and rewarding experience. Moreover, our findings also characterize the neural differences when perceiving and appreciating the naturalness and artificiality of landscapes from the visual perceptual encoding to the rewarding emotional experience and suggest the possibility that the inferior occipital lobe, the SPL, the fusiform gyrus, and the cuneus may be specifically associated with the perception and appreciation of landscape gardens. In addition, as 2‐D architectural photographs were used in this study, further research will be needed with 3‐D architectural images or virtual reality techniques to explore the appreciation of architecture.

## CONFLICT OF INTEREST

The authors declared that the research was conducted in the absence of any commercial or financial relationships that could be construed as a potential conflict of interest.

## AUTHOR CONTRIBUTIONS

Wei Zhang and Xianyou He designed the experiments and drafted the article; Xiaoxiang Tang revised this manuscript critically; Sizhe Liu contributed to data preprocessing and the revised version of the manuscript; Ting Li, Jinhui Li and Shuxian Lai contributed to data collection and making experimental materials.

## Data Availability

The data that support the findings of this study are available from the corresponding author upon reasonable request.
